# Supplementary material for: Predicting visual function by interpreting a neuronal wiring diagram
Source: Nature. 2024 Oct 2;634(8032):113–23. doi: 10.1038/s41586-024-07953-5 (PMC11446822; doi:10.1038/s41586-024-07953-5)
Supplement: Supplementary file 6 — CRF and ERF predictions for individual TmY4 and TmY9 cells. Analogous to Supplementary Data 3, but for TmY target types. Shown are the top four monosynaptic pathways, the strongest pathway passing through each of the top ten intermediary types (ranking from Extended Data Fig. 7), and the trisynaptic pathway Tm1–TmY–Dm3–TmY (see the section entitled Prediction of spatial normalization). [file 41586_2024_7953_MOESM6_ESM.zip › DataS4/TmY9q/720575940603842988.pdf]

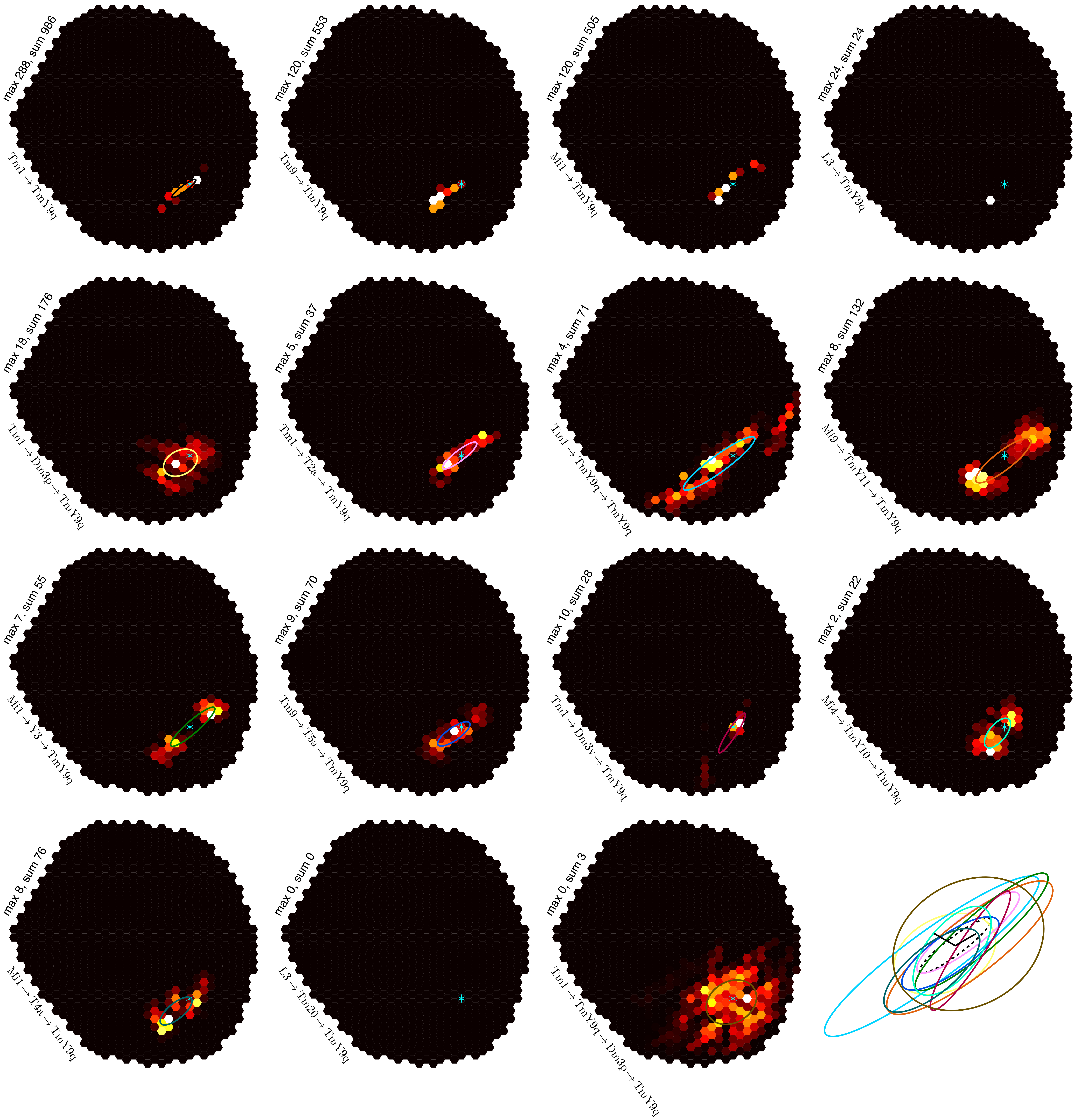
max 288, sum 986
Tm1 → TmY9q
max 120, sum 553
Tm9 → TmY9q
max 120, sum 505
Mi1 → TmY9q
max 24, sum 24
L3 → TmY9q
max 18, sum 176
Tm1 → Dm3p → TmY9q
max 5, sum 37
Tm1 → T2a → TmY9q
max 4, sum 71
Tm1 → TmY9q → TmY9q
max 8, sum 132
Mi9 → TmY11 → TmY9q
max 7, sum 55
Mi1 → Y3 → TmY9q
max 9, sum 70
Tm9 → T5a → TmY9q
max 10, sum 28
Tm1 → Dm3v → TmY9q
max 2, sum 22
Mi4 → TmY10 → TmY9q
max 8, sum 76
Mi1 → T4a → TmY9q
max 0, sum 0
L3 → Tm20 → TmY9q
max 0, sum 3
Tm1 → TmY9q → Dm3p → TmY9q
